# Epoxidation of Fatty Acid Methyl Esters Derived from Algae Biomass to Develop Sustainable Bio-Based Epoxy Resins

**DOI:** 10.3390/polym12102313

**Published:** 2020-10-10

**Authors:** Pamela Hidalgo, Simona Álvarez, Renato Hunter, Alejandra Sánchez

**Affiliations:** 1 Department of Industrial Processes, Faculty of Engineering, Universidad Católica de Temuco, Temuco 4780000, Chile; salvarez2011@alu.uct.cl (S.Á.); alsanbec@uct.cl (A.S.); 2 Department of Mechanical Engineering, Universidad de La Frontera, Casilla 54-D, Temuco 4811230, Chile; renato.hunter@ufrontera.cl

**Keywords:** bio-based resin, fatty acid methyl esters, in situ transesterification, microalgae, epoxidation

## Abstract

The objective of this research was to investigate the development of epoxides from *Chlorella vulgaris* lipids to obtain a novel bio-based resin. The process involved the production of fatty acid methyl esters (FAMEs) by in situ transesterification of microalgal biomass, followed by epoxidation of the FAMEs to obtain bioresin. During the FAME production process, an assessment was made of the main factors affecting the production of unsaturated fatty acid methyl esters (UFAMEs), such as catalyst dosage and methanol:hexane volume ratio. For step epoxidation, an evaluation of the catalyst concentration, temperature and formic acid:hydrogen peroxide ratio was made. From the results obtained, UFAME production was maximized using 20 wt% of catalyst dosage and a volume ratio of 1:2 (*v/v*, methanol:hexane). Then, in the epoxidation stage, a higher yield was obtained using 1 wt% of catalyst with a volume ratio of 1:1 and maintaining a temperature of 70 °C. The bioresin was blended with neat epoxy resin (DGEBA) and cured with tetraethylenepentamine (TEPA). Bio-based resin was characterized via Fourier transform infrared spectroscopy (FTIR), Raman spectroscopy, thermogravimetric analysis (TGA) and dynamic mechanical analysis (DMA) to evaluate this material as an alternative source for oleochemistry.

## 1. Introduction

Algae have been identified as a suitable source for the development of bioproducts, in part due to their not competing with food sources, their ability to grow on waste resources, high photosynthetic efficiency and high growth rate [1]. Different products have been obtained from algae, such as feedstock for biofuels, polymers with thermo-mechanical properties comparable in quality to synthetic polymers obtained from polysaccharides, oligosaccharides and lipids for the food, textile and manufacturing industries [2].

Microalgae are known to contain a high amount of proteins, lipids and carbohydrates, which could be the feedstock to produce polymeric materials and composite resins [3]. This could be a path to reduce dependence on the use of crude oil to develop petrochemically produced polymers. Moreover, in the last few years, there has been increased interest in the preparation of environmentally friendly polymers based on lipids, which are a suitable alternative to achieve a reduction of plastic accumulation in landfills and the resulting environmental hazard. The use of lipids for polymer production has been motivated mainly by the fact that they are abundant, renewable and relatively cheap [4].

In particular, *Chlorella* strains have been considered as a potential oleaginous model due to their high photosynthetic efficiency, growth rate and lipid productivity, as well as their easy cultivation. *Chlorella* strains have been widely studied for the production of fatty acids for use in biodiesel production [5]. Previous studies have displayed that *Chlorella vulgaris* can reach a lipid content of 40% depending on growth conditions [6] and have been considered one of the most promising candidates for commercial lipid production.

Lipids contain double bonds in their structure, which can be used in a wide range of reactions. Given the great potential of lipids, epoxidation is a useful tool for the production of bio-based epoxy resins. The most reported raw materials used for bio-based polymers are soybean oil, linseed oil and rapeseed oil [7,8,9], with carbon–carbon double bonds which can be converted into reactive functional groups via an epoxidation reaction [10]. In this context, microalgae lipids contain double bonds in their structure which enable them to be transformed, although this feature has been little studied to date.

Moreover, in the synthesis of biopolymers, not only lipids have been used as raw material, the use of their derivatives has also been gaining attention because of their excellent performance. Reports have been made of epoxidation using derivatives of lipids such as fatty acid methyl esters (FAMEs), used traditionally as a substitute for diesel due to the accessibility of their structure to undergo chemical modifications such as the transformation of double bonds into oxirane groups [11]. This is produced from the transesterification of lipids or oleaginous biomass by direct conversion, using a catalyst and an alcohol as an acyl-acceptor [12]. FAMEs have low viscosity and when mixed into the formulation, thus reducing the need for conventional organic solvents into the epoxidation reaction.

In this article, we report on a study of epoxidation of fatty acid methyl esters obtained from *Chlorella vulgaris* to develop sustainable bio-based epoxy resins. The fatty acid methyl esters were produced from in situ transesterification of microalgal biomass. In the process, the main factors affecting the reaction, such as catalyst dosage and methanol:hexane volume ratio, were evaluated. Then, in the epoxidation stage, to obtain epoxidized fatty acid methyl ester, a study of factors such as catalyst concentration, temperature and formic acid:hydrogen peroxide volume ratio was carried out. In addition, an analysis of the behavior of bio-based epoxy resins and neat epoxy resin was conducted.

## 2. Methodology

### 2.1. Reagents

The reagents and auxiliary materials used in the experimental work were formic acid (HCOOH, 85%), hydrogen peroxide (aqueous H_2_O_2_, 35%) and sulfuric acid (H_2_SO_4_, 95–98%) from Merck. Sodium bicarbonate (NaHCO_3_), hydrobromic acid (HBr), glacial acetic acid (CH_3_COOH) and anhydrous sodium sulfate (Na_2_SO_4_) came from Emsure^®^ (Merck KGaA, Santiago, Chile). Bisphenol A diglycidyl ether (DGEBA) came from Sigma-Aldrich, (Santiago, Chile) and tetraethylene pentamine (TEPA) came from Britez Ltd. (Santiago, Chile) The solvents methanol (MeOH), hexane (C_6_H_14_), heptane (C₇H₁₆) and chlorobenzene (C_6_H_5_Cl), from Sigma-Aldrich, were also used.

### 2.2. Experimental Setup and Procedure

#### 2.2.1. Production of Unsaturated Fatty Acid Methyl Esters (UFAME) by In Situ Transesterification of Microalgal Biomass

The experiments were performed in a screw-capped vessel (20 mL) containing the reaction mixtures and 1 g microalgae. *Chlorella vulgaris* microalgae were used in all experiments. Methanol was used as an acyl acceptor and sulfuric acid (H_2_SO_4_) was used as a catalyst. The reaction was maintained at 200 rpm for 4 h at 60 °C and then stopped by adding hexane (C_6_H_14_) and distilled water (3:2 *v*/*v*). The upper phase was separated and washed with a solution of sodium bicarbonate ((NaHCO_3_, 10%) and distilled water until a neutral pH of 7.0 was attained. After this, the organic phase was dried with anhydrous sodium sulfate (Na_2_SO_4_) and filtered. Finally, the solvent was evaporated by distillation and the liquid product was weighed and quantified as fatty acid methyl esters (FAMEs), which were subsequently analyzed via gas chromatography. Heptadecanoic acid methyl ester (C17:0) of chromatographic purity was used as an internal standard. FAME production was evaluated according to Hidalgo et al. (2015) [13]. Unsaturated fatty acid methyl ester (UFAME) content was obtained from the total FAME profile.

An experimental design of surface response methodology was applied to determine the influence of the operational variables on UFAME yield. The factorial design was performed using Design Expert 7.0 software. The independent variables used in this study were catalyst concentration (X_1_, wt%.) and the volume ratio of methanol:hexane (X_2_, *v*/*v*); UFAME yield (wt%.) was used as the response variable. The independent variables and levels of the experimental design are shown in Table 1 with coded and uncoded factors.

#### 2.2.2. Preparation of Epoxidized Unsaturated Fatty Acid Methyl Esters (EUFAME)

The extracted UFAME was homogenized with heptane (C₇H₁₆, co-solvent) using a 3:4 *v/v* heptane-to-UFAME ratio. The UFAME was then acidified with formic acid (HCOOH), and hydrogen peroxide (H_2_O_2_) was slowly added dropwise. The reaction was maintained for 4 h at a constant temperature and with continuous agitation. Chlorobenzene (C_6_H_5_Cl) at a 1:1 *v/v* ratio was added to separate the organic layer. Subsequently, the lower phase was separated and washed with sodium bicarbonate (NaHCO_3_, 10 wt%) and distilled water until reaching a neutral pH. The chlorobenzene was removed by distillation for the quantification of epoxidized UFAME (or EUFAME), corresponding to bio-based epoxy resin.

EUFAME, evaluated as a function of the oxygen oxirane content (OOC), was determined using a hydrobromic acid (HBr) solution in glacial acetic acid (CH_3_COOH) following American Oil Chemists’ Society (AOCS) standard test method Cd 9-57 [14]. EUFAME yield according to Hidalgo et al. (2019) [15] was evaluated.

For the epoxidation of UFAME, an experimental design of surface response methodology was used (as shown in Table 2). EUFAME yield was used as the response variable, and catalyst concentration (wt%), temperature and formic acid:hydrogen peroxide volume ratio were used as independent variables. The effects of these factors on the EUFAME value were assessed using the experimental matrix shown in Table 2.

#### 2.2.3. Preparation of Bio-Based Epoxy Blends

Bio-based epoxy blends were prepared using EUFAME, DGEBA and TEPA as the curing agent and 10 wt% of epoxidized UFAME was added to DGEBA. The mixture and curing agent were homogeneously mixed in 1:1 weight proportions and then stirred for 10 min. Subsequently, epoxy blends were poured into a silicone mold, cured at 50 °C for 8 h, and then post-cured at 65 °C for 12 h in an oven (Memmert UF-260 universal) and stored at room temperature.

### 2.3. Analytical Techniques

#### 2.3.1. Gas Chromatography

The identification and quantification of UFAME was performed via gaseous chromatography using a gaseous chromatograph coupled to a flame ionization detector (Clarus 600, Perkin Elmer, MA, USA). A capillary column, Elite-5 ms (30 m × 250 μm × 0.25 μm) with the following temperature program was used: 60 °C for 1 min, then the temperature increased at a rate of 30 °C/min up to 180 °C. After this, the temperature was increased at a rate of 2 °C/min up to 215 °C and finally raised to 240 °C for 6 min at a rate of 20 °C/min. Both the injector and detector temperature were maintained at 300 °C and 250 °C, respectively. The carrier gas used was He.

In the preparation of the injection vials, a 10 μL sample and 233 μL internal standard (methyl heptadecanoate, 2000 mg/mL) were used.

#### 2.3.2. FTIR Spectroscopy

The chemical structure was identified by FTIR spectroscopy (Cary 630 FT-IR, Agilent Technologies, CA, USA). The FT-IR spectra of the different samples were recorded at room temperature and between 600 to 4000 cm^−1^ frequency ranges.

#### 2.3.3. Raman Spectroscopy

Raman measurements were carried out using Raman spectroscopy (Dual LIBS-Raman, *Unchained Labs)*. The degree of crosslinking (α) was calculated as:

(1) $$\propto =100*\frac{I_{1275\;-}^{O}I_{1275}^{t}}{I_{1275\;}^{O}}$$

where $I_{1275\;}^{O}$ is the normalized intensity of the 1275 cm^−1^ peak at the beginning and $I_{1275}^{t}$ is the normalized intensity at time t. The peaks were normalized by dividing their intensities by that of the 1600 cm^−1^ peak corresponding to the phenyl ring [16].

#### 2.3.4. Thermogravimetric Analysis (TGA)

The thermal degradation of the bio-based epoxy blends were carried out using a thermogravimetric analyzer (TGA STA 6000, Perkin Elmer, MA, USA). Twenty milligrams of sample were heated from 20 to 600 °C at a 15 °C/min heating rate under a nitrogen atmosphere. The weight loss of the sample was recorded as a function of the temperature.

#### 2.3.5. Dynamic Mechanical Analysis (DMA)

The DMA measurements were performed using a dynamic mechanical analyzer (DMA 7e, Perkin Elmer, MA, USA) under a nitrogen atmosphere (20 mL/min), with heat increasing from 25 to 250 °C at 10 °C/min. A static force of 750 mN and a dynamic force of 700 mN at a frequency of 10 Hz were used. The samples for the DMA experiments were prepared in square shapes with average dimensions of 19 × 19 × 3 mm.

## 3. Results and Discussion

### 3.1. Evaluation of Reaction Conditions of UFAME Production

An experimental design of surface response methodology was used to study the interactive effects of critical variables of the transesterification process by direct conversion of microalgal biomass. According to the results shown in Table 1, UFAME yields varied from 3.9 to 58.8 (wt%) in the different combinations of variables of the experimental design. Table 3 shows the statistical analysis of variance (ANOVA) conducted to study the significance and the effects of significant individual terms and their interactions on the response, as well as multiple regression coefficients of optimization of the UFAME production process. Applying multiple regression analysis, the results were fitted to a polynomial equation. Thus, the mathematical regression model for UFAME yield in terms of coded factors was as follows:

(2) $$UFAME\;\left(\mathrm{wt}\%\right)=31.00+22.15\cdot X_{1}+5.3\cdot X_{2}+0.35\cdot X_{1}\cdot X_{2}$$

The larger F-values and smaller *p*-values are indicators of the significance of the model and its factors. According to ANOVA results, the prediction model was significant to *p*-value < 0.0001 and the F-value was higher than the critical value of 4.38. In terms of the independent variables, catalyst concentration (X_1_, wt%.) was a significant variable (*p*-value <0.05 and F-value > 4.38), presenting an important effect on the chemical conversion. The R-squared value of model prediction was 0.9647. This means that 96.47% of the total variation in UFAME yield is attributed to the evaluated experimental variables. Moreover, the adequate precision value was higher than 4, indicative of satisfactory model discrimination [17].

**Figure 1:**
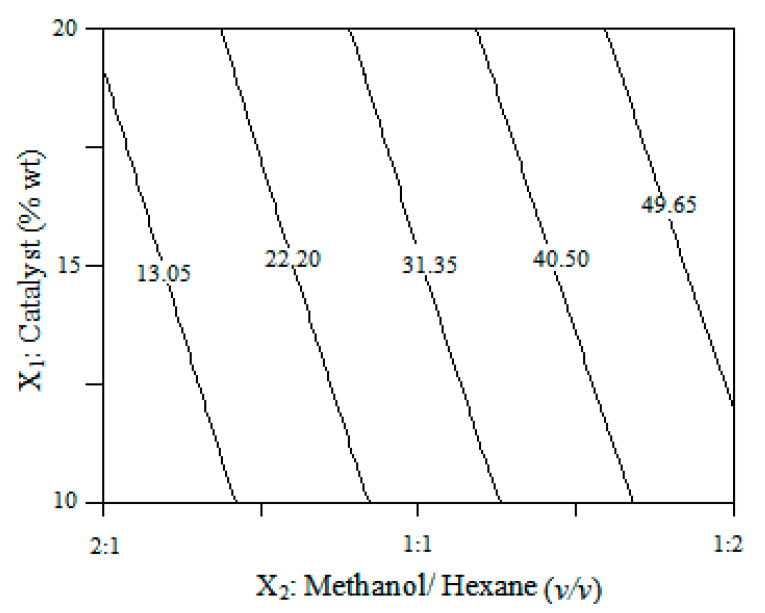
Contour plot for the effect of the catalyst (X_1_, wt) and methanol/hexane (X_2_, *v/v*) on the UFAME yield.

**Figure 2:**
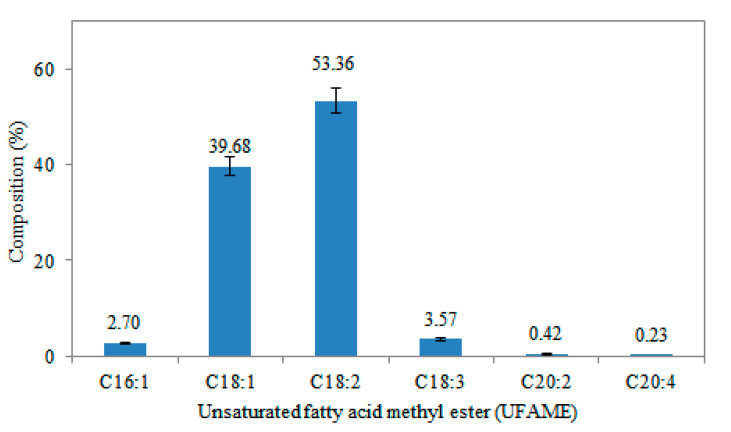
Unsaturated fatty acid methyl ester composition of *Chlorella vulgaris* microalgae. The bars indicate significant differences at *p* < 0.05.

The relationships between the parameters and UFAME yield were linear or almost linear. According to the model, the catalyst dosage has a positive effect on UFAME yield. During the reaction, the catalyst acts both on the lipid transesterification and on side reactions of the hydrolysis of protein and carbohydrates of cell constituents, consuming part of the catalyst originally available for the lipid transesterification [13]. A graphical representation of the regression equation (Equation (2)) is shown in Figure 1, presenting the effects of the tested variables (X_1_ and X_2_) on the response. According to the figure, an increment of the catalyst dosage improved UFAME yield. The optimal condition that maximizes UFAME production was determined from regression equations, corresponding to a value of 58.8% of UFAME yield using 20 wt% of catalyst dosage and a volume ratio of 1:2 (*v/v*, methanol:hexane). UFAME is mainly composed of linoleic acid methyl ester (C18:2) and oleic acid methyl ester (C18:1) (see Figure 2). The molecular weight obtained for UFAME is 270.99 g/mol.

**Figure 3:**
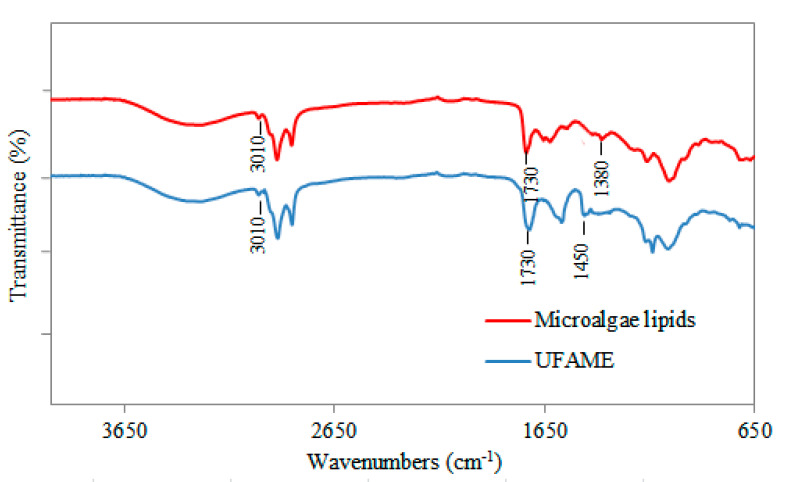
FT-IR spectrum of lipids and UFAME of *Chlorella vulgaris* microalgae.

The FT-IR spectra for the microalgae lipids and the UFAME obtained under optimal conditions are presented in Figure 3. Although few differences are observed, peaks appear in the region between 1800–1700 cm^−1^ that can be attributed to the stretching of C=O of the ester groups of lipids and UFAME. Moreover, the peak at 1450 cm^−1^ can correspond to an asymmetric stretching of -CH_3_ present in the UFAME spectrum and the peak at 1380 cm^−1^ can be attributed to O-CH_2_ groups in the glycerol of the lipids [18,19].

### 3.2. Evaluation of UFAME Epoxidation Reaction Conditions

The experimental design of surface response methodology was used to evaluate UFAME epoxidation reaction conditions. With regard to UFAME production, the optimal condition of the previous stage was implemented. The epoxidized UFAME (EUFAME) yield was evaluated using the colorimetric determination method [15].

The study of the significance and effect of significant individual terms and their interactions was obtained from the experimental design analysis. Table 4 shows the ANOVA and regression coefficients of the polynomial model. According to the ANOVA results, the prediction model proved to be adequate, establishing that the independent variables, temperature (X’_2_) and volume ratio of formic acid/hydrogen peroxide (X’_3_) were significant to a *p*-value of < 0.0001 and an F-value > 4.38. Moreover, the R-squared value showed that 96.47% of the total variation in oxygen oxirane content (OCC) yield is attributed to the evaluated experimental variables. The regression model for EUFAME yield in terms of coded factors is as follows:

(3) $$EUFAME=45.66+0.19\cdot X_{1}{}^{\prime }+6.81\cdot X_{2}{}^{\prime }-8.19\cdot X_{3}{}^{\prime }+2.84\cdot X_{1}{}^{\prime }\cdot X_{2}{}^{\prime }-2.99\cdot X_{2}{}^{\prime }\cdot X_{3}{}^{\prime }$$

According to the model, temperature (X’_2_) has a positive effect on the epoxidation reaction, leading to increased EUFAME yield. Conversely, the *volume ratio* of formic acid/hydrogen peroxide (X’_3_) displayed a negative effect, diminishing the EUFAME yield. The positive effect of the temperature was due to the speedier formation of performic acid with increased temperature, resulting in a faster epoxidation rate [20]. The negative effect of the formic acid/hydrogen peroxide volume ratio may be due to diminishing stability of the oxirane ring with the increase in peroxide hydrogen content [21]. Thus, an increase in the formic acid/hydrogen peroxide ratio from 1:1 to 1:3 vol/vol diminished the EUFAME yield, suggesting that experimental variables should be carefully controlled in order to reduce negative effects on EUFAME yield.

**Figure 4:**
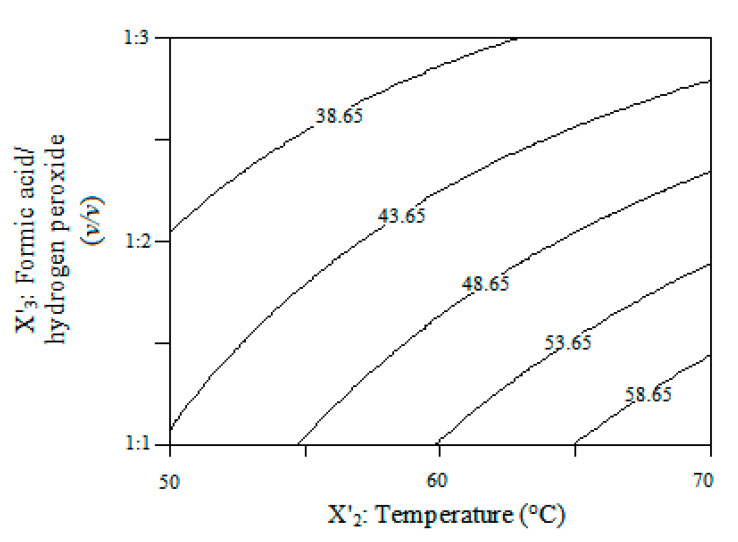
Contour plot for the effect of formic acid/hydrogen peroxide (X’_3_, *v/v*) and temperature (X’_2_, °C) on EUFAME yield.

**Figure 5:**
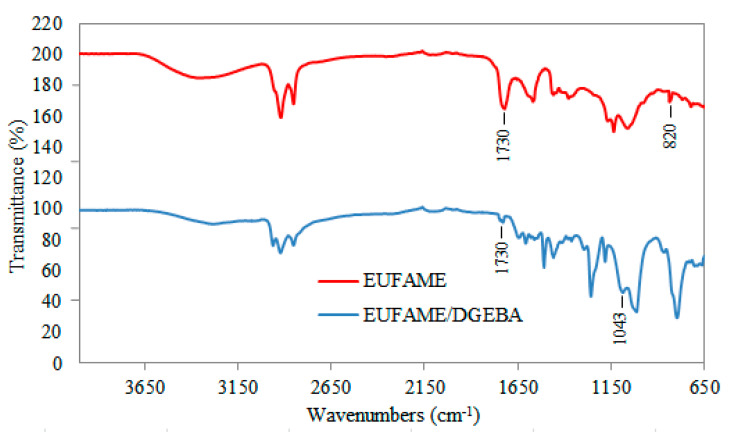
FT-IR spectra of uncured EUFAME and cured EUFAME/DGEBA. EUFAME: Unsaturated Fatty Acid Methyl Esters. EUFAME/DGEBA: Bio-based epoxy blend of EUFAME and DGEBA (neat epoxy resin).

Figure 4 is a graphical representation of the regression equation (Equation (3)) considering the significant variables of the predicted model for fixed parameters. As shown in Table 4, these variables caused a significant effect on the response. It can be observed that the epoxy value was higher at higher temperatures with a decrease in the formic acid/hydrogen peroxide volume ratio. Moreover, according to the predicted model, EUFAME yield is maximized (66.07%) in the production of bio-based epoxy resin using 1 wt% of catalyst with a volume ratio of 1:1 and maintaining a temperature of 70 °C. The obtained result (64.9%) was reasonably close to the predicted value, indicating that the model developed can be considered to be accurate and reliable. The molecular weight of EUFAME is 317.75 g/mol. The FT-IR spectra for the EUFAME are shown in Figure 5, where the results show that the peak corresponding to the double bond disappears. According to Figure 3, this peak is found in the absorption band located at 3010 cm^−1^. Moreover, peaks corresponding to epoxy groups appear at 820 cm^−1^ [15,22], confirming the synthesis of epoxides from UFAME in the production of bio-based epoxy resin. 

### 3.3. Characterization of Bio-Based Epoxy Blend

Bio-based epoxy blend was prepared from a mixture of EUFAME and DGEBA, with 10 wt% of EUFAME added to DGEBA. The changes in the functional groups due to crosslink networks cured with TEPA of the FT-IR spectrum are shown in Figure 5 (see EUFAME/DGEBA). According to the results, it was observed that the absorption band located at 820 cm^−1^ corresponding to the epoxide ring present in uncured EUFAME disappeared after the curing process. This confirms that the epoxide group reacts with zwitterions generated from the curing agent [23]. In the EUFAME/DGEBA spectrum, the band located at 1043 cm^−1^ corresponds to C-O-C groups of the crosslinked epoxy network [23,24]. With regard to the band located at around 1730 cm^−1^, an overlap of the peak of crosslinked EUFAME and C=O stretching of the EUFAME ester groups are observed.

**Figure 6:**
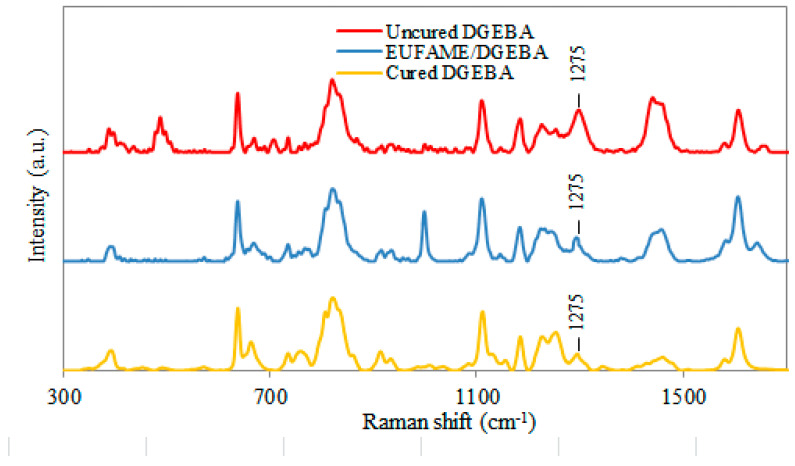
Raman spectra of EUFAME/DGEBA, DGEBA and uncured DGEBA.

In Figure 6, the Raman spectra of uncured DGEBA, cured DGEBA and the cured EUFAME/DGEBA blend are shown. A decrease in peak intensity at 1275 cm^−1^ corresponding to the epoxide group was observed in EUFAME/DGEBA and cured DGEBA. The degree of crosslinking of the epoxide group and the EUFAME/DGEBA blend by Raman spectroscopy is 87.21%, comparable to 93.01% of neat epoxy resin (cured DGEBA).

**Figure 7:**
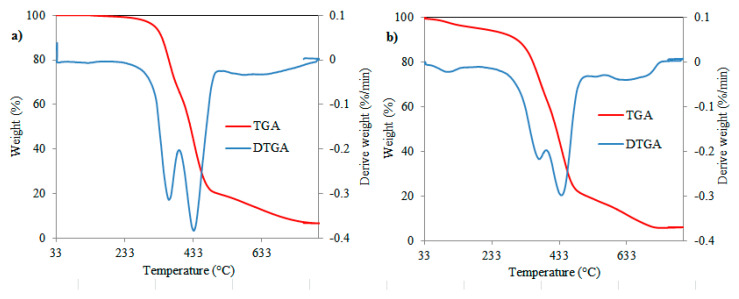
TGA curves for (**a**) cured neat epoxy resin (DGEBA) and (**b**) cured EUFAME/DGEBA blend.

Figure 7 shows the thermogravimetric analysis (TGA) and derivative thermogravimetric (DTG) curves of neat epoxy resin and the EUFAME/DGEBA blend. The temperatures corresponding to mass loss are presented in Table 5. In the TGA curve, the samples present a two-stage decomposition profile. The neat epoxy resin showed earlier onset degradation (T_onset_) at 310 °C and final degradation (T_endset_) at 523 °C with 4.46% char residue. In the case of the EUFAME/DGEBA blend, T_onset_ degradation begins at 302 °C and T_endset_ occurs at 512 °C with 4.51% char residue at 755 °C, indicating that the pyrolysis of the neat epoxy resin is similar to that of the EUFAME/DGEBA blend.

Moreover, the 5%, 10% and 50% weight loss thermal degradation (T_5_, T_10_, T_50_) of the EUFAME/DGEBA blend is obtained at 201 °C, 305.83 °C and 423.34 °C, respectively, whereas in DGEBA it occurs at 319.95 °C, 314.2 °C and 426.41 °C. Therefore, the results indicate that the lower thermal stability of the blend (EUFAME/DGEBA) could be due to a decrease in the crosslink density [11]. At the same time, it is possible to conclude that EUFAME exhibits lower thermal stability compared with DGEBA.

The thermal degradation rate of the EUFAME/DGEBA blend and neat epoxy are comparable, according to the DTGA curve in Figure 7a,b. A double peak corresponding to the maximum effect on the rate of degradation is found. The first peak degradation temperature (T_1_) appears at 369.56 °C and the second peak degradation temperature (T_2_) at 436.29 °C for the EUFAME/DGEBA blend, while in the neat epoxy, these occur at 360.66 °C and 433.22 °C, respectively.

**Figure 8:**
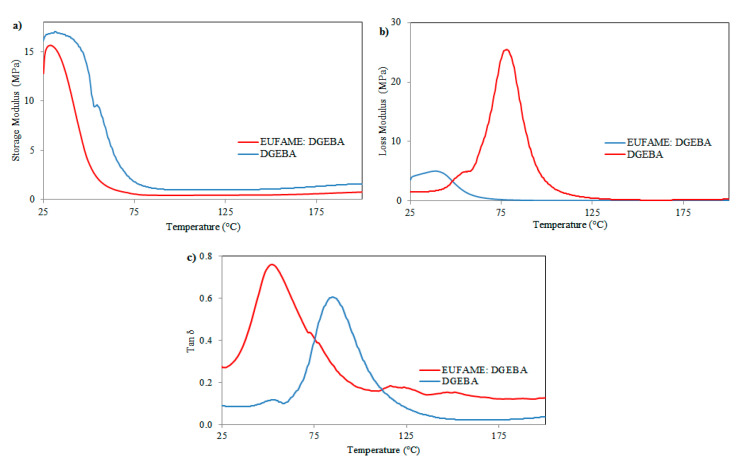
Variation in temperature of (**a**), storage modulus (**b**), loss modulus and (**c**) loss tangent (tan δ) of cured neat epoxy resin (DGEBA) and cured EUFAME/DGEBA blend.

The DMA results for the damping storage modulus (E’), loss modulus (E”) and factor (Tan δ) are shown in Figure 8a–c. The Tan δ results showed that the EUFAME:DGEBA blend exhibits a decrease in glass transition temperature (Tg) in comparison with neat epoxy resin, due to the presence of a long aliphatic chain. The Tg values of neat epoxy and the EUFAME:DGEBA blend were 83.02 °C and 53.01 °C, respectively. Moreover, in the EUFAME:DGEBA blend, the peak intensity of the Tan δ curve decreased and the curve became broader. This confirmed the presence of elastomeric components which can absorb a greater amount of energy [23].

The results of E’, E” and crosslink density are presented in Table 6. The results show a decrease with the incorporation of EUFAME, which can be attributed to a plasticization effect due to the flexibility of the long aliphatic chain in the backbone of EUFAME and lower reactivity [23,25].

The high E’ of the epoxy resin is attributed to the presence of bulky bisphenol groups in the polymeric networks and a higher degree of crosslinking. A non-abrupt fall in storage modulus is observed [23], unlike in the case of the EUFAME:DGEBA blend, where an abrupt fall in storage modulus occurs. It was found that the crosslink density of the EUFAME:DGEBA blend decreased, leading to a decrease in storage modulus as compared to neat epoxy resin.

## 4. Conclusions

In the synthesis of the bioresin, fatty acid methyl esters (FAMEs) were successfully produced by the in situ transesterification of microalgal biomass, followed by an epoxidation stage. From the results obtained, in the transesterification stage, the catalyst dosage had a significant effect on UFAME yield. From the results obtained, UFAME production was maximized using 20 wt% of catalyst dosage and a volume ratio of 1:2 (vol/vol, methanol:hexane). In the epoxidation of UFAME, the volume ratio of formic acid/hydrogen peroxide and temperature were significant. A higher yield was obtained using 1 wt% of catalyst with a volume ratio of 1:1 and maintaining a temperature of 70 °C. In the evaluation of the behavior of the bio-based epoxy resin blended with neat epoxy resin (DGEBA) and cured with tetraethylene pentamine (TEPA), a decrease in thermal stability was observed. Besides, a decrease in Tg due to an increase in flexibility and molecular chain movement occurred. Moreover, the DMA study reported that the ability to absorb energy increases upon the incorporation of EUFAME within DGEBA. According to the result observed in the Tanδ curve, this is due to a slight broadening of EUFAME/DGEBA. Although EUFAME/DGEBA showed a decrease in mechanical, cross-linking and thermal properties compared to the analogous material from neat resin, the produced materials were notably more ductile. Besides, the use of EUFAME was proved to cause no significant sacrifice in properties in comparison to neat resin when it was used a 10 wt% formulation. The study concludes that epoxidized fatty acid methyl esters obtained from *Chlorella vulgaris* could be a potential candidate to develop sustainable bio-based epoxy resins and partially replace neat epoxy resin.

## Figures and Tables

**Table 1:** Experimental design of the effects of independent variables on UFAME yield.

Run	Catalyst (wt%) X_1_	Methanol/Hexane (*v*/*v*) X_2_	UFAME Yield (wt%)
**1**	20 (1)	1:2 (1)	58.8
**2**	15 (0)	1:1 (0)	25.5
**3**	15 (0)	1:1 (0)	29.7
**4**	15 (0)	1:1 (0)	21.0
**5**	15 (0)	1:1 (0)	18.3
**6**	15 (0)	1:1 (0)	23.0
**7**	10 (−1)	2:1 (−1)	3.9
**8**	20 (1)	2:1 (−1)	47.5
**9**	10 (−1)	1:2 (1)	13.8

**Table 2:** Experimental design of the effects of independent variables on the EUFAME value.

Run	Catalyst (wt%) X’_1_	Temperature (°C) X’_2_	Formic Acid/Hydrogen Peroxide (*v/v*) X’_3_	EUFAME Yield (%)
**1**	0.5 (−1)	70 (1)	1:1 (−1)	62.4
**2**	1 (1)	70 (1)	1:1 (−1)	64.9
**3**	1 (1)	70 (1)	1:3 (1)	46.1
**4**	0.75 (0)	60 (0)	1:2 (0)	49.5
**5**	0.5 (−1)	50 (−1)	1:1 (-1)	46.3
**6**	0.5 (−1)	70 (1)	1:3 (1)	36.5
**7**	1 (1)	50 (−1)	1:3 (1)	30.6
**8**	0.5 (−1)	50 (−1)	1:3 (1)	36.7
**9**	1 (1)	50 (−1)	1:1 (−1)	41.8

**Table 3:** ANOVA of the microalgae biomass transesterification model.

Source	Coefficient Estimate ^a^	Sum of Squares	df	Mean Square	F-Value	*p*-Value
Model		2076.07	3	692.02	36.29	0.0023 ^c^
Intercept	31					
X_1_: Catalyst (wt%)	22.15	1962.63	1	1962.6	102.91	0.0005 ^b^
X_2_: Methanol/Hexane (*v/v*)	5.3	112.99	1	112.99	5.92	0.0717
X_1 × 2_	0.35	0.44	1	0.44	0.023	0.8863
Curvature		124.8	1	124.8	6.54	0.0628
Pure error		76.28	4	19.07		
Total		2277.15	8			
R-squared	0.9647					
Adeq Precision	16.9					

^a^ Coefficients refer to the model given. ^b^ Significant at level *p* < 0.001. ^c^ Significant at *p* < 0.05.

**Table 4:** ANOVA of the UFAME epoxidation model.

Source	Coefficient Estimate ^a^	Sum of Squares	df	Mean Square	F-value	*p*-value
Model		1043.66	5	208.73	31.52	0.031 ^b^
Intercept	45.66					
X’_1_: Catalyst (wt%)	0.19	0.28	1	0.28	0.042	0.8558
X’_2_: Temperature (°C)	6.81	371.28	1	371.28	56.07	0.0174 ^b^
X’_3_: Formic acid/hydrogen peroxide (*v/v*)	−8.19	536.28	1	536.28	80.99	0.0121 ^b^
X’_1_X’_2_	2.84	64.41	1	64.41	9.73	0.0892
X’_2_X’_3_	−2.99	71.4	1	71.4	10.78	0.0816
Curvature		13.09	1	13.09	1.98	
Residual		13.24	2	6.62		
Total		1069.99	8			
R-squared	0.9875					

^a^ Coefficients refer to the model given. ^b^ Significant at *p* < 0.05.

**Table 5:** Thermal degradation behaviors for cured neat epoxy resin (DGEBA) and cured EUFAME/DGEBA blend.

Sample	T_onset_ (°C) ^a^	T_endset_ (°C) ^b^	T5 (°C) ^c^	T10 (°C) ^d^	T50 (°C) ^e^	T_1_ (°C) ^f^	T_2_ (°C) ^g^	Residue at 755 °C (%) ^h^
DGEBA	310	523	319.95	314.2	426.41	360.66	433.22	4.46
EUFAME:DGEBA	302	512	201.1	305.83	423.34	369.56	436.29	4.51

^a^ Initial degradation temperature (°C). ^b^ Final degradation temperature (°C). ^c^ 5% weight loss temperature (°C). ^d^ 10% weight loss temperature (°C). ^e^ 50% weight loss temperature (°C). ^f^ First peak degradation temperature (°C). ^g^ Second peak degradation temperature (°C). ^h^ Residue (wt%) at 755 °C.

**Table 6:** DMA values for cured neat epoxy resin (DGEBA) and cured EUFAME/DGEBA blend.

Sample	Tg (°C)	E´ (MPa) ^a^	E” (MPa) ^a^	Crosslink Density (× 10^3^ mol/m^3^) ^b^
DGEBA	83.02	17.47	4.46	1.84
EUFAME:DGEBA	53.01	15.55	1.51	1.10

^a^ Evaluated at 30 °C. ^b^ Evaluated according to Sudha et al. (2017) [26].
